# Free water–mediated associations among choroid plexus enlargement, white matter lesions, and cognitive performance in type 2 diabetes mellitus

**DOI:** 10.3389/fendo.2026.1838132

**Published:** 2026-06-18

**Authors:** Ling Li, Peichun Pan, Jing Li, Min Tang, Dongsheng Zhang, Xiaoyan Lei, Xiaoling Zhang, Jie Gao

**Affiliations:** 1 Department of MRI, Shaanxi Provincial People’s Hospital, Xi’an, Shaanxi, China; 2 Department of Graduate, Shaanxi University of Chinese Medicine, Xianyang, Shaanxi, China; 3 Department of Graduate, Xi’an Medical University, Xi’an, Shaanxi, China

**Keywords:** choroid plexus, cognition, free water, type 2 diabetes mellitus, white matter

## Abstract

**Background:**

Recent studies indicate choroid plexus (CP) enlargement and glymphatic-related brain fluid changes in type 2 diabetes mellitus (T2DM), but how these alterations relate to white matter hyperintensities (WMH) and cognitive impairment in T2DM remains unclear.

**Methods:**

This study comprised a total of 104 patients diagnosed with T2DM along with 56 healthy controls, who underwent brain MRI and neuropsychological assessment. CP volume, free water (FW) fraction, perivascular space (PVS) volume, and WMH volumes were quantified. Within the T2DM group, the relationship among imaging markers, WMH burden and cognitive performance was examined using multivariable linear regression and mediation analyses.

**Results:**

The T2DM patients exhibited lower Symbol Digit Modalities Test (SDMT) scores and larger CP volume, FW fraction, PVS volume, and periventricular WMH (PWMH) volume than controls (all *P* < 0.05). In T2DM patients, CP volume (β = 0.20, *P* = 0.01) and FW fraction (β = 0.29, *P* < 0.01) were independently associated with PWMH, while FW fraction (β = −0.31, *P* < 0.01) and PWMH (β = −0.32, *P* = 0.01) were independently linked to SDMT. Mediation analyses showed significant statistical indirect effects through FW in the CP–PWMH association (β = 0.07, 95% CI 0.01 to 0.13), through PWMH in the FW–SDMT association (β = −0.09, 95% CI −0.18 to −0.02), and through FW in the CP–SDMT association (β = −0.07, 95% CI −0.15 to −0.02).

**Conclusions:**

FW-related brain fluid alterations are associated with CP enlargement, periventricular white matter lesion burden, and processing speed performance in T2DM.

## Introduction

Globally, Type 2 diabetes mellitus (T2DM) is among the commonest metabolic disorders in the world (1). Beyond its traditional vascular and metabolic complications, accumulation of evidence suggests that T2DM is associated with neurological abnormalities, including neurophysiological and structural brain alterations, as well as cognitive impairments affecting attention, executive function, and processing speed (2, 3). The precise pathological processes underlying T2DM-associated cognitive decline remain poorly understood but is likely complex and multifactorial, involving chronic hyperglycemia, insulin resistance, micro- and macrovascular damage, neuroinflammation, oxidative stress, and vascular dysfunction (4, 5).

Brain alterations are common in patients with T2DM, and white matter changes are especially well characterized. An increasing number of studies have reported greater burden of white matter hyperintensities (WMHs) in individuals with T2DM (6, 7). Lower cognitive performance in T2DM and other age-related conditions has also been associated with WMH burden (8–10). Greater WMH burden in T2DM has been associated with metabolic stress, cerebral hypoperfusion, vascular injury, breakdown of blood-brain barrier (BBB) and inflammatory processes (11). However, the biological processes associated with these white matter changes and their relationship with cognitive dysfunction, remain to be fully elucidated.

Recently, increasing attention has been directed toward the glymphatic system, which plays a critical role in the regulation of brain fluid and clearance of waste products, and may be implicated in white matter vulnerability and cognitive decline. MRI markers sensitive to brain fluid and perivascular alterations, such as perivascular spaces (PVS), free water (FW) in the extracellular space, and diffusion-based indices, including the analysis along the perivascular space (ALPS) index, have been suggested as potential indirect indicators of fluid changes affecting the glymphatic system and perivascular clearance (12–14). Researchers have indicated that glymphatic-related markers have been increasingly associated with cognitive decline in vascular, neurodegenerative, and metabolic diseases (15–19). Recent studies have reported increased PVS and decreased ALPS index in T2DM (20, 21), suggesting potential disruption of glymphatic-related brain fluid dynamics. Nonetheless, direct *in vivo* evidence linking glymphatic-related fluid changes with white matter lesion load and cognition in T2DM is scarce.

The choroid plexus (CP) is centrally involved in brain fluid homeostasis, serving as the primary site of cerebrospinal fluid (CSF) production and an important site of immune surveillance and molecular transfer between the blood and the CSF (22). Beyond these functions, the CP may also serve as a neuroendocrine-relevant interface between systemic metabolic status and the brain, participating in the exchange and signaling of insulin-related molecules, glucocorticoids, and other circulating hormones that may influence CSF composition and brain homeostasis. Thus, in T2DM, CP alterations may reflect not only neuroinflammatory or fluid-regulatory processes but also interactions between metabolic dysregulation and central neuroendocrine signaling. By virtue of its location at the blood–CSF interface and its permeable microvasculature, the CP provides a route for the entry of immune cells and soluble mediators into the brain. Furthermore, the CP has been implicated in the regulation of neuroinflammatory pathways and brain aging (23). Postmortem studies and *in vivo* imaging of neurodegenerative and neuroinflammatory diseases have shown molecular and morphological CP alterations; therefore, CP enlargement may serve as a potential imaging marker for neuroinflammation and disturbed brain fluid regulation (24–26). A recent MRI study in patients with T2DM reported CP enlargement and observed a statistical indirect association involving CP volume in the relationship between cognitive impairment and insulin resistance (27). Nonetheless, that investigation did not evaluate the MRI markers related to glymphatic function or white matter lesion burden. CP morphology, glymphatic-related MRI markers, white matter lesion burden, and cognitive performance in T2DM have not yet been systematically characterized.

Building on the aforementioned considerations, we hypothesize that in individuals with T2DM, CP morphology would be associated with glymphatic-related fluid alterations, WMH burden, and cognitive performance, and that these imaging markers would show statistical indirect associations with one another. To test this, we performed a cross-sectional study with T2DM patients and matched healthy controls, integrating quantitative CP volumetry, glymphatic-related MRI markers (FW fraction, PVS volume, and the ALPS index), and automated WMH segmentation within a multimodal imaging framework. Our goals were to (1) identify group differences in CP volume, glymphatic-related MRI markers, WMH burden, and cognitive performance; (2) investigate associations among CP volume, glymphatic-related MRI markers, WMH burden, and cognitive performance in the T2DM group; and (3) examine whether glymphatic-related MRI markers showed cross-sectional statistical indirect effects in the associations among CP morphology, WMH burden, and cognitive performance.

## Materials and methods

Conducted as a prospective cross-sectional study, this research adhered strictly to the principles outlined in the Helsinki Declaration and its subsequent amendments. The study received ethical approval from the Institutional Ethics Committee of Shaanxi Provincial People’s Hospital (No. 2022K101). Prior to their inclusion in the study, all participants provided written informed consent.

### Participants

Patients with T2DM were consecutively recruited from both the outpatient clinics and inpatient wards of the Department of Endocrinology at Shaanxi Provincial People’s Hospital between January 2022 and December 2024. Age-, sex-, and education-matched healthy controls (HCs) were sought from the local community via advertisement. The selected participants were all between the ages of 40 and 70 years, were right-handed, and had completed a minimum of six years of formal education. The T2DM group also must meet these criteria: (1) diagnosed with T2DM according to the 2022 American Diabetes Association standards (28); (2) duration of diabetes ≥ 1 year; and (3) stable antidiabetic treatment and glycemic control (diet, oral drugs, and/or insulin injections) for at least three months before inclusion. Additional inclusion criteria for the HC group were: (1) no history of diabetes or prediabetes; and (2) fasting plasma glucose and HbA1c within the normal range at screening. For all participants, the exclusion criteria were: (1) history of transient ischemic attack, stroke, or other major neurological disorders (for example, traumatic brain injury, brain tumors, epilepsy, vascular malformations, multiple sclerosis and Parkinson’s disease); (2) diagnosis of dementia or major psychiatric disorder; (3) severe systemic disease (for example, end-stage renal, hepatic, or cardiac failure); (4) alcohol or substance abuse; (5) MRI contraindications; (6) inability to complete the neuropsychological assessments and MRI examinations, or MRI images of insufficient quality for analysis; and (7) documented diagnosis of sleep disorders, including obstructive sleep apnea or chronic insomnia, or regular use of medications known to substantially affect sleep or arousal, based on medical history and medication records. Demographic and clinical data, including vascular risk factors, diabetes duration, glycemic indices, sleep-related medical history, and medication use, were collected using structured interviews and medical records.

### Neuropsychological assessment

A series of neuropsychological evaluations were administered to all participants by neurologists who were formally trained in these assessments and were blinded to both group allocation and imaging data. The Mini-Mental State Examination (MMSE) served as a general measure of cognitive abilities, while the Symbol Digit Modalities Test (SDMT) and the Color Trails Test, Part 1 (CTT-1) specifically evaluated processing speed and attention. The Color Trails Test, Part 2 (CTT-2) was employed to evaluate executive functioning, and the Rey Auditory Verbal Learning Test (RAVLT) was utilized to measure episodic verbal memory. The Clock Drawing Test (CDT) evaluated visuospatial abilities. The Beck Depression Inventory (BDI) was used to assess depression.

### MRI acquisition

All MRI examinations were conducted using a 3.0T MR scanner (Ingenia, Philips Medical Systems, the Netherlands) fitted with a 16-channel phase-array head coil. MRI scans were conducted between 8:00 and 12:00 to minimize variability in glymphatic measurements associated with circadian rhythms and sleep wake cycle. The imaging protocol comprised sagittal 3D T1-weighted imaging (3D T1WI), axial T2-weighted imaging (T2WI), axial 3D T2-weighted fluid-attenuated inversion recovery (T2-FLAIR), and axial diffusion-weighted imaging. Sequence parameters were as follows: 3D T1WI: repetition time (TR) = 10 ms, echo time (TE) = 4.6 ms, inversion time (TI) = 400 ms, slice thickness = 1 mm, field of view (FOV) = 256 × 256 mm, matrix = 256 × 256; T2WI: TR = 2,500 ms, TE = 80 ms, slice thickness = 6 mm, matrix = 328 × 254; 3D T2-FLAIR: TR = 4,800 ms, TE = 340 ms, slice thickness = 1 mm, FOV = 256 × 256 mm, matrix = 256 × 256; diffusion-weighted imaging: 32 directions, b value = 0, 1,000 s/mm^2^, TR/TE = 6,000/150 ms, slice thickness = 2 mm, FOV = 256 × 256 mm, matrix = 128 × 128, number of excitations = 1.

### Imaging processing

#### CP segmentation and volume quantification

Structural 3D T1WI images were processed using FreeSurfer 7.2.0 (https://surfer.nmr.mgh.harvard.edu/) to obtain CP volumes. The standard recon-all pipeline was run to generate automated subcortical segmentation (ASEG). For every subject, we extracted the left and right CP labels from the ASEG output. The absolute CP volume in cubic millimeters was obtained by summing the left and right CP volumes. The total intracranial volume (ICV) was derived from the FreeSurfer-estimated total intracranial volume (eTIV). To account for variations in head size, the normalized CP volume was calculated by dividing the absolute CP volume by the ICV. The normalized CP volume was used in all subsequent statistical analyses, including ANCOVA, partial correlation, regression, and mediation analyses. All automated CP segmentations were visually checked for anatomical plausibility, and only segmentations of acceptable quality were used in the final analyses.

#### Free water mapping

A regularized bi-tensor FW model from the Diffusion Imaging in Python (DIPY) package (https://dipy.org/) was employed to obtain FW fraction maps from the preprocessed diffusion-weighted images, separating the diffusion signal into tissue and isotropic FW components (29). Given the potential influence of inter-subject misregistration and partial volume contamination in voxelwise diffusion analyses, the tract-based spatial statistics (TBSS) pipeline was used to project FW maps onto a white matter skeleton. By restricting statistical testing to the centers of major white matter tracts, this method enhances anatomical specificity and reduces residual alignment variability across subjects. Each subject’s fractional anisotropy (FA) map was nonlinearly registered to the FMRIB58_FA standard-space template (1 × 1 × 1 mm) using FMRIB Software Library (FSL). The mean FA image was then skeletonized to form a white matter skeleton, representing the centers of major white matter fiber tracts, with an FA threshold of 0.20 to exclude gray matter and CSF. Using FA-derived transformations, individual FW maps were warped into the same standard space and then projected onto the common FA skeleton. Mean FW fraction values were extracted from the skeletonized white matter for statistical analysis, wherein a higher FW value was represented as increased extracellular free water content and potential interstitial fluid dysregulation. All registrations and skeleton projections were visually inspected to ensure adequate alignment.

#### Perivascular space quantification

PVS masks were automatically segmented on 3D T1WI using a previously described pipeline based on Advanced Normalization Tools (ANTs), FSL, and the Quantitative Imaging Toolkit (QIT) (30). Briefly, bias field correction and spatial normalization were performed with ANTs, followed by skull stripping in FSL to remove nonbrain tissue. Afterward, both linear and nonlinear transformations were used to align the images to the 1-mm^3^ MNI152 template. White matter tissue was segmented with FMRIB’s Automated Segmentation Tool implemented in the FSL to provide a brain tissue mask. After removal of noise using nonlocal means filter technique with QIT to increase signal-to-noise ratio, Frangi filter was applied to enhance tubular structures to bring out PVS-like structures. To construct whole-brain PVS masks, the filtered images were binarized with a threshold (t = 2.3) that was determined empirically and has already been tested (31, 32). The total PVS volume (mm³) within the supratentorial brain was calculated and normalized to intracranial volume. Quality control protocol was applied to all PVS masks. An experienced rater reviewed the segmentation outputs to assess their anatomical plausibility and registration accuracy. Parameters relating to the segmentation were adjusted iteratively and the images were reprocessed until the PVS masks were acceptable.

#### DTI-ALPS index

The semi-automated ROI-based method proposed by Taoka et al. (14) was used to calculate the DTI-ALPS index. The diffusion-weighted images were preprocessed routinely, including nonbrain skull stripping, denoising, correction for Gibbs ringing artifacts, and adjustments to account for distortions induced by susceptibility, eddy currents, and head motion. Fractional anisotropy (FA) and diffusivity maps along the x, y, and z axes (D_x_, D_y_, and D_z_) were obtained by fitting diffusion tensors. Each subject’s FA map was nonlinearly aligned with the JHU-ICBM FA template, and this transformation was then applied to the D_x_, D_y,_ and D_z_ maps. Spherical ROIs (5 mm) were manually placed on bilateral projection and association fibers at the lateral ventricle bodies. The superior corona radiata and superior longitudinal fasciculus were identified as projection and association fibers using the JHU-ICBM-DTI-81 atlas. A single experienced rater, trained in this protocol and blinded to all clinical information as well as group assignment status, performed ROI placement. After visual inspection, the ROIs were slightly adjusted to avoid enlarged lateral ventricular spaces or subcortical lesions, and were then applied to the diffusivity maps (D_x_, D_y_, and D_z_). We extracted diffusivities along the x-, y-, and z-axes from these ROIs. The ALPS index was calculated as (D_xproj_ + D_xassoc_)/(D_yproj_ + D_zassoc_). ALPS indices for left and right hemispheres were computed separately, and their mean values were used for further analyses.

#### White matter lesion segmentation

WMHs were segmented by applying the UBO Detector pipeline (33) to 3D T2-FLAIR and T1WI. The UBO Detector applies an automated, intensity-based clustering algorithm, combined with spatial priors and a built-in training set, to generate probabilistic lesion maps in native space. These probability maps were thresholded at a lesion probability of 0.7 and subjected to morphological cleanup to obtain binary WMH masks. Lesions within 10 mm of the ventricular surface were classified as periventricular WMHs (PWMH), whereas those located farther are termed deep WMHs (DWMH). PWMH and DWMH volumes were normalized by ICV. A well-trained rater, who was unaware of the clinical data, visually inspected all WMH segmentations produced by the UBO Detector. Cases with gross misclassification or registration errors were reprocessed with adjusted parameters or excluded from further analyses.

### Statistical analysis

Statistical analysis was conducted by using IBM SPSS Statistics version 22.0 (IBM Corp., Armonk, NY, USA). The Shapiro–Wilk test assessed normality of variables. Differences between groups were evaluated using t-tests or Mann–Whitney U tests for continuous variables and χ² tests for categorical variables, as applicable. ANCOVA was used to compare group differences in normalized CP volume, FW fraction, ALPS index, PVS volume, and WMH volume, as well as cognitive scores, with adjustments made for age, sex, and years of education. To explore the relationships between normalized CP volume and glymphatic-related metrics (FW fraction, ALPS index, and PVS volume) within the T2DM group, partial correlation analyses were conducted controlling for age, sex, and years of education. Additional partial correlation analyses were performed to examine the associations of HbA1c and fasting blood glucose with the main imaging and cognitive variables, as well as the association between SDMT and CTT-1. The Benjamini–Hochberg method was used to adjust for multiple comparisons where appropriate. Multiple linear regression models assessed independent associations of imaging markers with white matter hyperintensity burden and cognitive performance. Because SDMT was the only cognitive measure that survived FDR correction in the between-group comparison, SDMT was carried forward as the main exploratory cognitive outcome for downstream regression and mediation analyses. To evaluate whether imaging–cognition associations extended to other cognitive domains, the same regression framework was additionally applied to non-SDMT cognitive measures, including CTT-1, CTT-2, RAVLT immediate recall, RAVLT delayed recall, and CDT. In all regression analyses, three sequentially adjusted regression models were applied. Model 1 adjusted for demographic variables (age, sex, and years of education). Model 2 additionally adjusted for body mass index and the presence of vascular risk factors (hypertension, hyperlipidemia, and current smoking). Model 3 additionally adjusted for diabetes-related variables, including insulin use, disease duration, HbA1c, and fasting blood glucose. Because normalized PWMH volume showed a right-skewed distribution, an additional sensitivity analysis was performed using log-transformed normalized PWMH volume [ln(normalized PWMH volume × 10^4^ + 1)] to evaluate the robustness of PWMH-related ANCOVA and regression findings. A *post-hoc* sensitivity power analysis was conducted for the multivariable regression models to estimate the minimum detectable effect size based on the final T2DM analytic sample, α = 0.05, and 80% power. Mediation analyses were conducted within the T2DM group as exploratory analyses of statistical indirect effects among normalized CP volume, FW fraction, WMH burden, and SDMT performance. These mediation models were tested using PROCESS Model 4 with 5,000 bootstrap resamples and bias-corrected 95% confidence intervals (CIs), adjusted for age, sex, and years of education. Indirect effects were considered statistically significant when the 95% bootstrap confidence interval did not include zero. These models were interpreted as cross-sectional statistical indirect-effect analyses rather than evidence of temporal or causal pathways. For all other two-tailed statistical tests, significance was set at P < 0.05.

## Results

### Demographic and clinical characteristics

**Figure 1:**
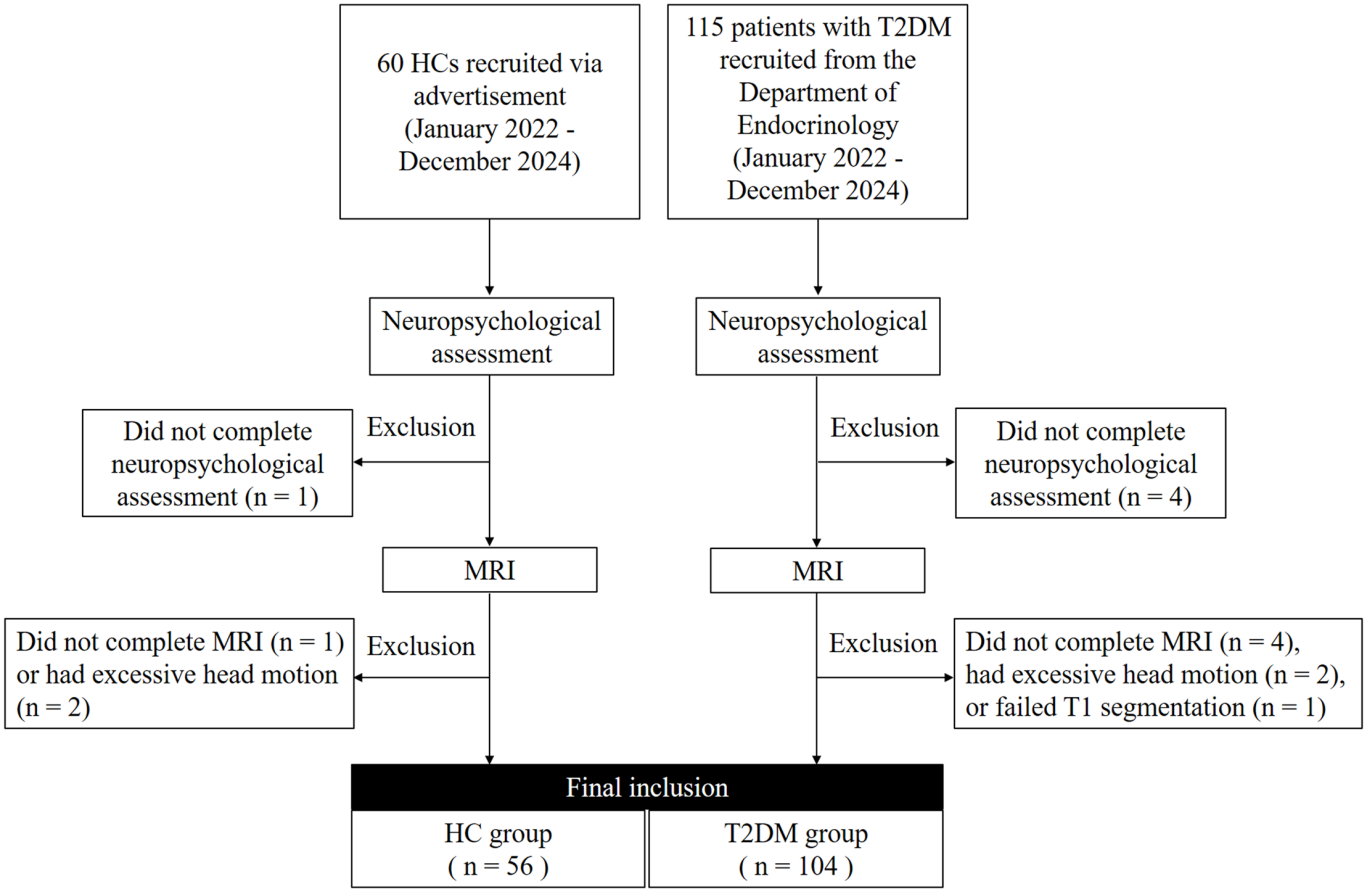
Flowchart of participant recruitment and inclusion.

A total of 104 patients with T2DM and 56 HCs met the eligibility criteria and were included in the final analysis. **Figure 1** summarizes the recruitment and selection process, while **Table 1** details demographic and clinical characteristics. By design, the two groups were comparable in age, sex, and years of education (all *P* > 0.05). As expected, T2DM patients showed significantly higher fasting glucose and HbA1c levels compared with HCs (both *P* < 0.001). No significant differences in hypertension, hyperlipidemia, and current smoking were found between the two groups (all *P* > 0.05).

**Table 1:** The demographic and clinical characteristics of the enrolled participants.

Clinic information	HC (n = 56)	T2DM (n = 104)	T/Z/F/χ2 value	P value
Age (years)	53.57 ± 6.46	54.69 ± 7.68	-0.93#	0.35
Male (n/%)	37/66.07%	76/73.07%	0.86&	0.35
Formal education (years)	13.05 ± 3.31	13.60 ± 2.94	-1.07#	0.29
BMI (kg/m2)	24.32 ± 2.62	25.21 ± 3.01	-1.86#	0.07
Fasting glucose (mmol/L)	5.13 ± 0.54	8.44 ± 3.42	-7.18#	<0.001*
HbA1c (%)	5.58 ± 0.47	8.11 ± 1.96	-12.51#	<0.001*
Hypertension (n/%)	14/25.00%	27/25.96%	0.02&	0.89
Hyperlipodemia (n/%)	20/35.71%	32/30.77%	0.41&	0.52
Smoking (n/%)	16/28.57%	32/30.77%	0.08&	0.77
Diabetes duration (years)	–	7.75 ± 6.25	–	–
Insulin use (n/%)	–	40/38.46%	–	–
MMSE	27.96 ± 2.42	28.35 ± 1.39	0.83	0.36
SDMT	44.52 ± 10.53	39.98 ± 9.14	9.13†	<0.01*
CTT-1 (s)	53.30 ± 29.07	56.92 ± 28.21	0.47†	0.50
CTT-2 (s)	165.48 ± 69.10	170.38 ± 53.23	0.13†	0.72
RAVLT immediate	41.68 ± 10.53	42.49 ± 8.61	0.34†	0.56
RAVLT delay	8.29 ± 2.99	8.64 ± 2.69	0.68†	0.41
CDT	24.21 ± 6.46	25.64 ± 5.96	2.25†	0.14
BDI	2(0, 5)	2(1, 6)	-0.22$	0.83

BMI, body mass index; HbA1c, glycated hemoglobin; MMSE, Mini-Mental State Examination; CTT-1, Color Trails Test part 1; CTT-2, Color Trails Test part 2; CDT, clock drawing test; RAVLT, Rey Auditory Verbal Learning Test; SDMT, Symbol Digit Modalities Test; BDI, Beck Depression Inventory. #T, independent samples t-test. $Z, Mann-Whitney U test. †F, one-way analysis of covariance; &χ2, chisquare test. Normally distributed data are presented as mean ± standard deviation; non-normally distributed data as median (first quartile, third quartile); categorical variables as n (%). *P < 0.05.

The patients with T2DM scored lower on the SDMT than HCs after adjusting for age, sex, and years of education (*P* < 0.01), whereas no significant between-group differences were observed for MMSE, CTT-1, CTT-2, RAVLT, CDT, or BDI scores. After Benjamini–Hochberg FDR correction for multiple comparisons, SDMT remained the only cognitive test with a significant between-group difference (*q* = 0.04). Thus, SDMT was carried forward as the main exploratory cognitive outcome for subsequent regression and mediation analyses.

### Group differences in imaging markers

**Figure 2:**
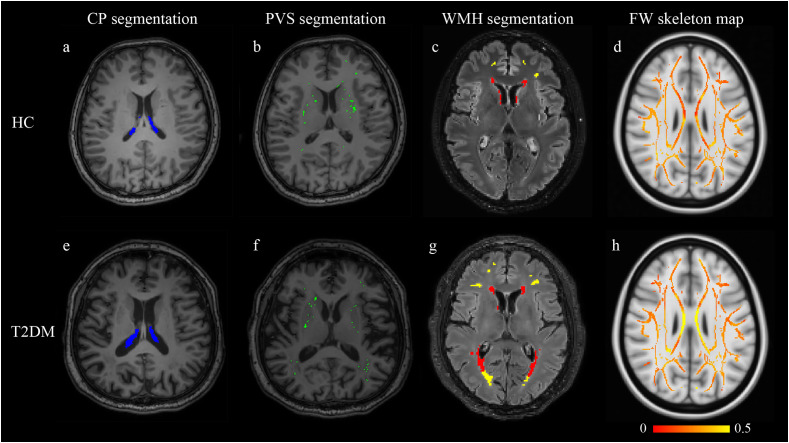
Representative MRI images showing segmentation and mapping results in one patient with T2DM (a 60-year-old male with 12 years of education) and one matched healthy control (a 58-year-old male with 12 years of education). The upper row shows the healthy control and the lower row shows the patient with T2DM. All images are fully anonymized and are shown as representative examples of segmentation quality and anatomical correspondence. From left to right, the panels show: **(a, e)** axial 3D T1-weighted images with choroid plexus (CP) segmentation overlaid in blue; **(b, f)** axial T1-weighted images with perivascular space (PVS) segmentation overlaid in green; **(c, g)** axial FLAIR images with periventricular white matter hyperintensity (PWMH) segmentation overlaid in red and deep white matter hyperintensity (DWMH) segmentation overlaid in yellow; and **(d, h)** free-water (FW) fraction projected onto the white matter skeleton and displayed using a red-to-yellow color scale representing FW fraction values from 0 to 0.5.

**Figure 3:**
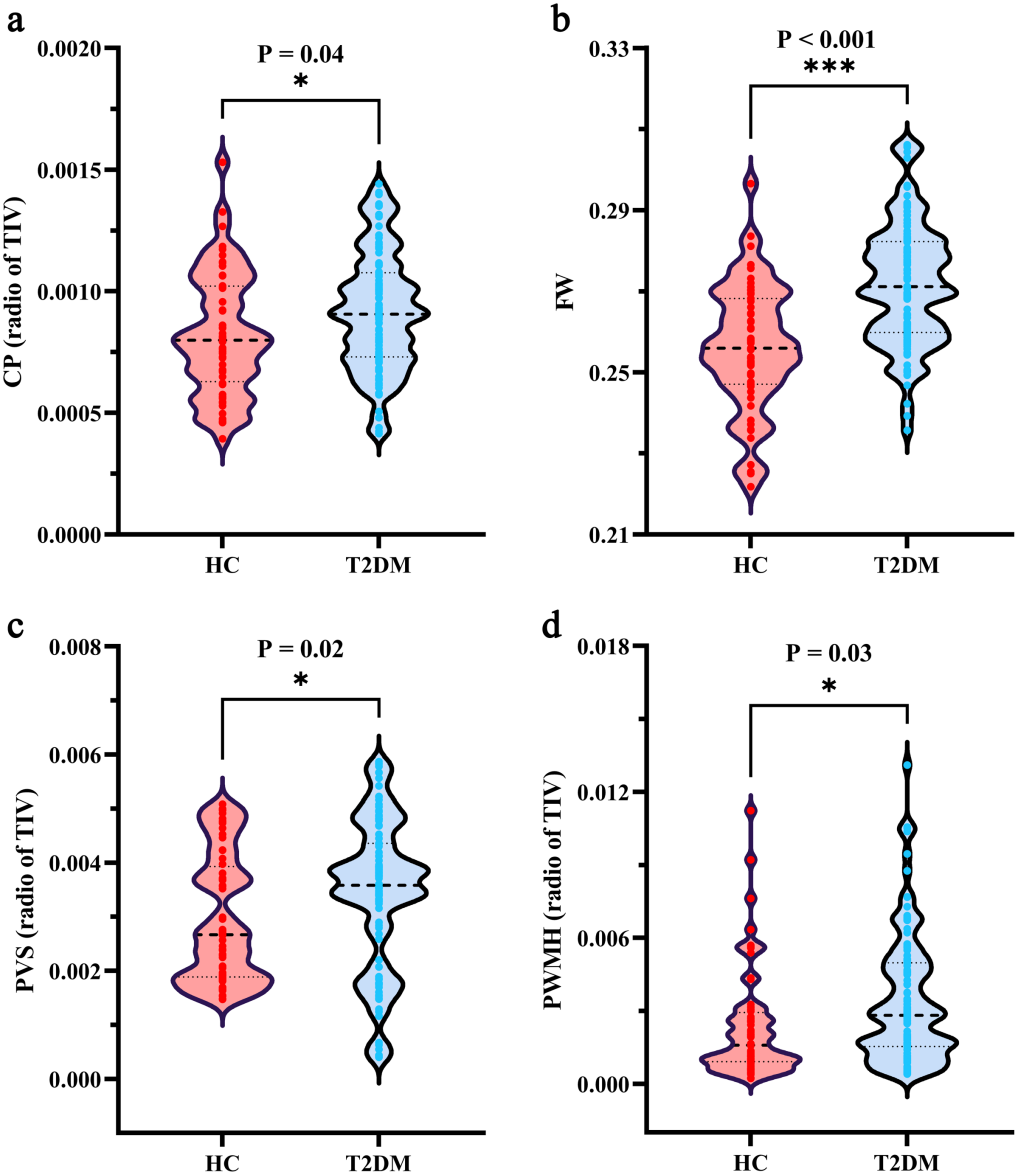
Differences in imaging markers between the HC and T2DM groups. Violin plots show the distribution of **(a)** normalized choroid plexus volume (CP volume/TIV), **(b)** free water (FW) fraction on the white matter skeleton, **(c)** normalized perivascular space volume (PVS volume/TIV), and **(d)** normalized periventricular white matter hyperintensity volume (PWMH volume/TIV) in HCs and patients with T2DM. Dashed lines indicate group means, and dotted lines indicate group medians. Group differences were tested using an analysis of covariance (ANCOVA), with age, sex, and years of education as covariates. Corresponding *P* values are displayed above the panels. Asterisks denote statistically significant differences (*P < 0.05; ***P < 0.001).

Representative MRI images from one matched HC and one patient with T2DM, illustrating CP segmentation, PVS segmentation, WMH segmentation, and FW skeleton mapping, are shown in **Figure 2**. After adjustment for age, sex, and years of education, patients with T2DM exhibited significantly larger normalized CP volume (F = 4.26, *P* = 0.04), higher FW fraction on the white matter skeleton (F = 23.13, *P* < 0.001), larger PVS volume (F = 4.61, *P* = 0.02), and greater PWMH volume (F = 4.71, *P* = 0.03) compared with HCs. These imaging outcomes remained significant after FDR correction (*q*-value range 0.003–0.04). In contrast, neither the ALPS index nor DWMH volume showed statistically significant between-group differences after adjustment for age, sex, and years of education. However, both measures showed small trend-level effects: the ALPS index tended to be lower in the T2DM group than in HCs (1.60 ± 0.19 vs. 1.66 ± 0.19; F = 3.72, P = 0.06, partial η² = 0.02), whereas DWMH volume tended to be higher in the T2DM group than in HCs (0.0016 ± 0.0013 vs. 0.0011 ± 0.0013; F = 3.71, P = 0.06, partial η² = 0.02). These trend-level findings did not survive correction for multiple comparisons. **Figure 3** shows violin plots that present the between-group comparisons for imaging measures.

### Partial correlation analyses

**Figure 4:**
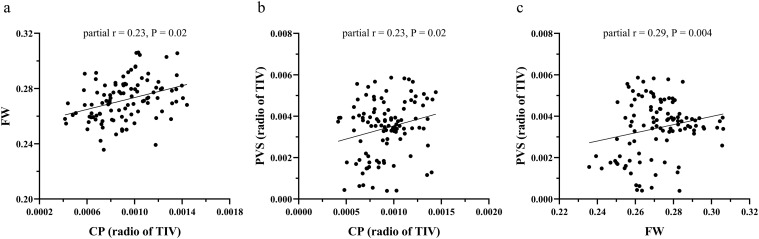
Partial correlations between choroid plexus volume and glymphatic-related imaging markers in patients with T2DM. The scatter plots illustrate the following relationships: **(a)** between normalized choroid plexus volume (CP) and free water (FW) fraction on the white matter skeleton, **(b)** between normalized CP volume and normalized perivascular space volume (PVS), and **(c)** between FW fraction and normalized PVS volume. Partial correlation coefficients (partial r) and corresponding *P* values (adjusted for age, sex, and years of education) are shown at the top of the panels.

Partial correlation analyses revealed positive correlations between normalized CP volume and FW fraction on the white matter skeleton (partial r = 0.23, *P* = 0.02) and PVS volume (partial r = 0.23, *P* = 0.02) after controlling for age, sex, and years of education. PVS volume was also positively associated with the FW fraction (partial r = 0.29, *P* < 0.01). All three correlations remained significant after FDR correction (all *q* ≤ 0.02; **Figure 4**). In contrast, the ALPS index did not show significant or trend-level associations with normalized CP volume, PVS volume, and FW fraction (all *P* > 0.05).

Additional partial correlation analyses were performed to examine the associations of glycemic indices with the main imaging and cognitive variables. HbA1c was not significantly associated with normalized CP volume, PVS volume, PWMH volume, DWMH volume, FW fraction, or SDMT performance after adjustment for age, sex, and years of education. Fasting blood glucose was positively associated with FW fraction (partial r = 0.22, P = 0.03), but was not significantly associated with normalized CP volume, PVS volume, PWMH volume, DWMH volume, or SDMT performance.

Because both SDMT and CTT-1 are processing-speed-related measures, we further examined their partial correlation within the T2DM group. After adjustment for age, sex, and years of education, SDMT was not significantly correlated with CTT-1 (partial r = 0.14, P = 0.17).

### Multivariable regression analyses

Linear regression analyses revealed that normalized CP volume (Model 1, β = 0.23, *P* < 0.01; Model 2, β = 0.20, *P* = 0.01; Model 3, β = 0.20, *P* = 0.01) and FW fraction (Model 1, β = 0.29, *P* < 0.01; Model 2, β = 0.24, *P* = 0.01; Model 3, β = 0.29, P < 0.01) were independently associated with PWMH volume. PVS volume was not associated with PWMH in Model 1 (β = 0.15, *P* = 0.07), but showed a modest independent association after further adjustment for vascular risk factors in Models 2 and 3 (both β = 0.18, *P* = 0.02). The PVS volume was independently associated with DWMH volume in all models (Model 1, β = 0.31, *P* < 0.001; Model 2, β = 0.33, *P* < 0.001; Model 3, β = 0.33, *P* < 0.001), whereas normalized CP volume was not significantly associated with DWMH (all *P* > 0.90). The association of the FW fraction with DWMH was weaker, being significant in Model 1 (β = 0.23, *P* = 0.03) and Model 3 (β = 0.25, *P* = 0.04), but not in Model 2 (β = 0.20, *P* = 0.07). The detailed regression coefficients and model fit statistics are presented in **Table 2**. Sensitivity analyses using log-transformed normalized PWMH volume are reported in **Supplementary Table 1**. These analyses showed that the PWMH group difference and PVS/FW–PWMH associations were relatively robust, whereas the CP–PWMH association was attenuated after log transformation.

**Table 2:** Linear regression analyses of imaging markers associated with white matter hyperintensity burden in patients with T2DM.

Parameter	Model 1			Model 2			Model 3		
	B (95% CI)	β	P	B (95% CI)	β	P	B (95% CI)	β	P
PWMH									
CP	2.50 (0.74, 4.26)	0.23	< 0.01*	2.19 (0.46, 3.93)	0.20	0.01*	2.20 (0.46, 3.98)	0.20	0.01*
FW	0.05(0.02, 0.08)	0.29	< 0.01*	0.04 (0.01, 0.08)	0.24	0.01*	0.05 (0.02, 0.09)	0.29	< 0.01*
PVS	0.28 (-0.02, 0.58)	0.15	0.07	0.35 (0.05, 0.65)	0.18	0.02*	0.35 (0.05, 0.65)	0.18	0.02*
DWMH									
CP	0.06 (-0.93, 1.04)	0.01	0.91	-0.03 (-1.01, 0.95)	-0.01	0.95	0.05 (-0.95, 1.06)	0.01	0.92
FW	0.02 (0.00, 0.04)	0.23	0.03*	0.02 (0.00, 0.04)	0.20	0.07	0.02 (0.00, 0.04)	0.25	0.04*
PVS	0.30 (0.13, 0.47)	0.31	< 0.001*	0.32 (0.15, 0.49)	0.33	< 0.001*	0.32 (0.15, 0.49)	0.33	< 0.001*

Model 1 was adjusted for age, sex, and years of education. Model 2 was further adjusted for body mass index and the presence of non-diabetes vascular risk factors. Model 3 additionally included diabetes-related variables, including insulin use (yes/no), diabetes duration (years), HbA1c, and fasting blood glucose. SDMT, Symbol Digit Modalities Test; CP, ICV-normalized choroid plexus volume calculated as absolute bilateral CP volume divided by intracranial volume and entered as the raw CP/ICV ratio without additional scaling; PVS, perivascular space; FW, free water; PWMH, periventricular white matter hyperintensity; DWMH, deep white matter hyperintensity; CI, confidence interval; β, standardized regression coefficient. *P < 0.05.

Linear regression analyses revealed that FW fraction (Model 1, β = −0.31, *P* < 0.01; Model 2, β = −0.32, *P* < 0.01; Model 3, β = −0.27, *P* = 0.01) and PWMH volume (Model 1, β = −0.28, *P* = 0.03; Model 2, β = −0.30, *P* = 0.02; Model 3, β = −0.28, *P* = 0.03) were independently associated with lower SDMT scores across all three models, whereas normalized CP volume was not independently associated with SDMT. **Table 3** summarizes the regression findings for SDMT.

**Table 3:** Linear regression analyses of imaging markers associated with SDMT performance in patients with T2DM.

Parameter	Model 1			Model 2			Model 3		
	B (95% CI)	β	P	B (95% CI)	β	P	B (95% CI)	β	P
CP	-1658.35 (-7952.30, 4635.60)	-0.04	0.60	-1935.06 (-8228.29, 4358.17)	-0.05	0.54	-1546.46 (-7892.78, 4799.86)	-0.04	0.63
FW	-189.47 (-309.75, -69.20)	-0.31	< 0.01*	-198.36 (-319.23, -77.48)	-0.32	< 0.01*	-167.25 (-297.483, -37.01)	-0.27	< 0.01*
PVS	-318.32 (-1397.18, 760.54)	-0.05	0.56	-140.06 (-1246.86, 966.74)	-0.02	0.80	-259.34 (-1369.15, 850.47)	-0.04	0.64
PWMH	-964.25 (-1809.82, -118.68)	-0.28	0.03*	-1022.66 (-1891.24, -154.08)	-0.30	0.02*	973.93 (-1864.42, -83.44)	-0.28	0.03*
DWMH	891.89 (-624.28, 2408.05)	0.13	0.25	754.45 (-781.90, 2290.80)	0.11	0.33	795.09 (-725.48, 2315.66)	0.12	0.30

Model 1 was adjusted for age, sex, and years of education. Model 2 was further adjusted for body mass index and the presence of non-diabetes vascular risk factors. Model 3 additionally included diabetes-related variables, including insulin use (yes/no), diabetes duration (years), HbA1c, and fasting blood glucose. SDMT, Symbol Digit Modalities Test; CP, ICV-normalized choroid plexus volume calculated as absolute bilateral CP volume divided by intracranial volume and entered as the raw CP/ICV ratio without additional scaling; PVS, perivascular space; FW, free water; PWMH, periventricular white matter hyperintensity; DWMH, deep white matter hyperintensity; CI, confidence interval; β, standardized regression coefficient. *P < 0.05.

Exploratory regression analyses were further performed using non-SDMT cognitive measures as outcomes, including CTT-1, CTT-2, RAVLT immediate recall, RAVLT delayed recall, and CDT. Normalized CP volume, FW fraction, PVS volume, PWMH volume, and DWMH volume were entered simultaneously as imaging predictors, with adjustment for age, sex, and years of education. These analyses did not reveal consistent associations between the imaging markers and these non-SDMT cognitive outcomes (**Supplementary Table 2**).

### Post-hoc sensitivity power analysis

The *post-hoc* sensitivity power analysis indicated that, with n = 104, α = 0.05, power = 0.80, and 8–10 predictors/covariates, the minimum detectable effect size for the multivariable regression models was f² = 0.15–0.17, corresponding to R² = 0.13–0.15. These values indicate that the study had reasonable sensitivity to detect moderate overall regression effects.

### Mediation analyses

Based on the results of multivariable regression analyses, cross-sectional mediation analyses were conducted to examine whether FW fraction and PWMH showed statistical indirect effects in the associations among normalized CP volume, WMH burden, and cognitive performance.

**Figure 5:**
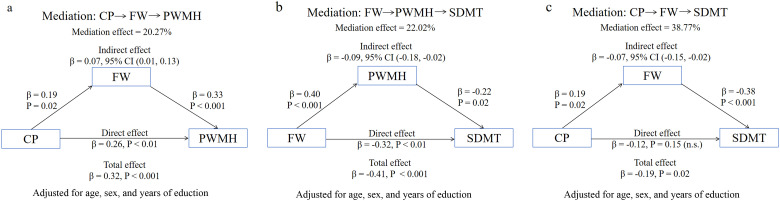
Cross-sectional mediation analyses within the T2DM group examining statistical indirect effects among normalized choroid plexus volume (CP), free water (FW) fraction, periventricular white matter hyperintensity (PWMH) burden, and symbol digit modalities test (SDMT) score in patients with T2DM. All displayed path coefficients and indirect effects were adjusted for age, sex, and years of education, and estimated using 5,000 bootstrap resamples with bias-corrected 95% confidence intervals (CI). **(a)** statistical indirect effect for the CP–PWMH association through FW fraction. **(b)** statistical indirect effect for the FW–SDMT association through PWMH burden. **(c)** statistical indirect effect for the CP–SDMT association through FW fraction.

Cross-sectional mediation analyses showed a significant statistical indirect effect of normalized CP volume on PWMH burden through FW fraction [standardized indirect effect β = 0.07, 95% CI (0.01, 0.13), mediation effect 20.27%] (**Figure 5a**).

Similarly, a significant statistical indirect effect was observed for the association between FW fraction and SDMT score through PWMH burden [standardized indirect effect β = −0.09, 95% CI (−0.18, −0.02), mediation effect 22.02%] (**Figure 5b**).

A significant statistical indirect effect was also observed for the association between normalized CP volume and SDMT performance through FW fraction [standardized indirect effect β = −0.07, 95% CI (−0.15, −0.02), mediation effect 38.77%] (**Figure 5c**). The direct association between normalized CP volume and SDMT performance was not statistically significant after accounting for FW fraction; however, this pattern should not be interpreted as evidence of complete mediation given the cross-sectional design, modest effect size, and relatively wide confidence interval of the indirect effect.

## Discussion

This cross-sectional multimodal MRI study identified a convergent pattern of brain alterations in patients with T2DM, including larger CP volume, increased FW fraction on the white matter skeleton, greater PVS volume, greater PWMH, and selective slowing of information processing speed as assessed by the SDMT compared with HCs. Within the T2DM group, CP volume was positively correlated with FW fraction and PVS burden, while FW fraction was closely linked to PVS burden. In multivariable regression models, CP volume and FW fraction were independently associated with PWMH burden, whereas FW fraction and PWMH burden were independently associated with lower SDMT scores. Cross-sectional mediation analyses further characterized the statistical association pattern among CP enlargement, FW fraction, PWMH burden, and processing speed performance. Specifically, significant statistical indirect effects were observed for the CP–PWMH association through FW fraction, for the FW–SDMT association through PWMH burden, and for the CP–SDMT association through FW fraction. Collectively, these findings support a hypothesis-generating associative framework in which CP enlargement, fluid-related microstructural abnormalities, periventricular WMH burden, and processing speed performance are interrelated in T2DM.

Our glymphatic-related imaging findings suggest a pattern of fluid-related white matter vulnerability in T2DM. The elevated FW fraction is generally considered to be a sensitive marker of extracellular water accumulation and edema-like changes and may reflect altered interstitial fluid dynamics or neuroinflammation (34–36). In our cohort, FW fraction was strongly associated with the PWMH burden and weakly with the DWMH, indicating its close association with small-vessel-disease-related white matter alterations (35, 37, 38). In parallel, PVS volume was enlarged in T2DM and correlated with FW fraction and WMH measures, which is in keeping with the concept that macroscopic dilation of the PVS may be associated with altered perivascular drainage and interstitial fluid overload (19, 39, 40). In particular, PVS volume showed a modest independent association with PWMH only after further adjustment for vascular risk factors, whereas it was robustly associated with DWMH in all models, suggesting a regionally heterogeneous relationship between fluid-related imaging markers and WMH phenotypes. The emergence of a modest PVS–PWMH association after adjustment for vascular risk factors may reflect a suppression or context-dependent adjustment effect, in which vascular risk covariates may have accounted for variance not directly related to the PVS–PWMH relationship, thereby allowing the independent association to become more apparent. However, the stronger and more consistent association between PVS volume and DWMH suggests that PVS burden may be more closely linked to deep white matter lesion vulnerability than to periventricular lesions.

This preferential PVS–DWMH association deserves further consideration. Whereas PWMH is anatomically adjacent to the ventricular and CSF–white matter interface and may be more closely related to CP-related inflammatory signaling, altered CSF-interface regulation, and diffuse extracellular water accumulation, DWMH occurs in deep white matter territories supplied by long penetrating arterioles. In these regions, enlarged PVS may more directly reflect impaired perivascular drainage, arteriolosclerosis, endothelial dysfunction, or interstitial fluid stagnation along deep medullary vessels. Thus, the differential associations of CP volume and FW fraction with PWMH, and PVS volume with DWMH, suggest a regionally heterogeneous pattern of white matter vulnerability in T2DM, with a periventricular CSF-interface-related pattern and a deeper perivascular drainage-related pattern.

In contrast to FW fraction and PVS volume, the ALPS index showed no significant group difference and was not significantly correlated with CP volume, PVS volume, or FW fraction within the T2DM group. ALPS was included because it is a widely used ROI-based diffusion MRI metric proposed to indirectly reflect perivascular directional diffusivity and provides a complementary glymphatic-related measure distinct from FW fraction and PVS volume. Therefore, the ALPS findings should be interpreted as informative rather than simply negative. Although the ALPS index tended to be lower in the T2DM group than in HCs, this difference did not reach statistical significance after adjustment for age, sex, and years of education. This direction is broadly consistent with Hu et al., who reported significant ALPS reductions associated with cognitive impairment in T2DM. The weaker ALPS effect in our study may reflect differences in disease stage and cognitive phenotype. Hu et al. focused on T2DM patients with cognitive impairment, whereas our cohort excluded participants with dementia and major neurological disorders and showed relatively selective SDMT slowing rather than broad neuropsychological impairment. Thus, ALPS abnormalities may be more detectable in older, more cognitively impaired, or more advanced T2DM populations, whereas FW fraction may be more sensitive to earlier or more diffuse extracellular fluid and neuroinflammatory microstructural alterations, raising the possibility of stage-dependent sensitivity among glymphatic-related MRI markers. Methodological factors may also contribute to the discrepancy. Unlike FW fraction, which was extracted from the white matter skeleton and may better reflect diffuse extracellular free water burden across major white matter tracts, ALPS is an ROI-based directional diffusivity ratio. Therefore, ALPS estimation is more dependent on diffusion acquisition, preprocessing, local fiber orientation, and ROI placement. The ROIs used for ALPS calculation are typically placed near the lateral ventricular body in projection and association fibers, including the superior corona radiata and superior longitudinal fasciculus. These periventricular regions are vulnerable to white matter lesions and partial-volume effects from adjacent CSF spaces. Therefore, the increased PWMH burden in the T2DM group may have introduced additional variability in ALPS estimation, even though visible lesions and enlarged ventricles were avoided where possible. Taken together, the dissociation between FW and ALPS indicates that these markers are not interchangeable and may capture different spatial scales and physiological components of brain fluid regulation in T2DM. This divergence highlights differences in construct validity and sensitivity between the two markers: FW may better index diffuse extracellular fluid-related white matter alterations, whereas ALPS may be more sensitive to localized perivascular directional diffusivity and more vulnerable to ROI-related methodological variability. Future longitudinal studies using lesion-informed ALPS protocols are needed to determine whether ALPS abnormalities become more prominent in later-stage or more cognitively impaired T2DM populations.

One important finding of this study is that FW fraction showed significant statistical indirect-effect patterns linking CP volume, periventricular WMH burden, and processing speed performance. Earlier research has associated CP enlargement with WMH burden and cognitive issues in cerebral small vessel disease (CSVD), supporting the biological plausibility of CP-related inflammatory and CSF-interface involvement (41). In our analyses, the FW fraction showed a statistical indirect effect in the association between CP volume and PWMH volume, which suggests that CP enlargement, FW fraction, and periventricular tissue vulnerability are statistically interrelated. These associations are biologically compatible with CP-related processes, such as altered CSF secretion or turnover, barrier permeability, and inflammatory signaling, but cannot establish their temporal or causal ordering. This is consistent with prior evidence showing associations between CP enlargement, WMH burden, and glymphatic-related alterations (42), and with studies reporting associations between inflammatory CSF markers, FW fraction and WMH burden in older individuals (43). In addition, FW fraction showed a significant statistical indirect effect in the association between CP volume and SDMT performance, suggesting that FW-indexed microstructural or fluid-related abnormalities statistically accounted for part of the association between CP volume and processing speed performance. However, the nonsignificant direct effect after accounting for FW fraction should not be interpreted as evidence of complete mediation, because the total CP–SDMT association was modest and the bootstrap confidence interval for the indirect effect was relatively wide, indicating uncertainty in the magnitude of the indirect association. Although causal inference cannot be made, the observed pattern is biologically plausible and hypothesis-generating, suggesting that CP morphology, CSF–immune interface alterations, fluid-related white matter microstructure, and cognitive performance may be interrelated. Longitudinal studies are required to determine the temporal ordering among CP enlargement, FW alterations, WMH progression, and cognitive decline.

In our cohort, SDMT was the only cognitive measure that remained significantly different between patients with T2DM and HCs after FDR correction, suggesting a relatively selective vulnerability of information processing speed. The SDMT evaluates the speed of information processing and attentional efficiency, and is known for its sensitivity to the integrity of white matter. In our cohort with T2DM, lower processing speed performance on the SDMT was consistent with the cognitive profile often related to CSVD (44). Previous neuroimaging research on diabetic patients has found links between a higher burden of WMH or white matter abnormalities and reduced processing speed (6, 45). These findings agree with our finding of inverse correlations between FW fraction and SDMT scores, as well as an independent association between PWMH burden and diminished SDMT performance. These results underscore an association among diabetes-related small vessel disease markers, white matter abnormalities, and processing speed performance (41, 42). In contrast, the lack of significant independent associations between SDMT performance and PVS burden suggests that macroscopic PVS dilation may show weaker cross-sectional associations with cognitive performance at this disease stage compared to FW-indexed microstructural abnormalities and WMH burden (46). After incorporating HbA1c and fasting blood glucose into the revised fully adjusted models, the main associations of CP volume, PVS volume, and FW fraction with WMH burden, as well as the associations of FW fraction and PWMH burden with SDMT performance, remained significant. Although fasting blood glucose was positively correlated with FW fraction, these findings suggest that the observed imaging and imaging–cognition associations were not simply explained by single-time-point glycemic indices.

The discrepancy between SDMT and CTT-1 may reflect differences in task demands and sensitivity. Although both measures are related to processing speed, they are not interchangeable. SDMT requires rapid visual scanning, sustained attention, symbol–number matching, and graphomotor output within a fixed time window, and may be particularly sensitive to distributed white matter network integrity. In contrast, CTT-1 is a timed visual search and sequencing task that may be more influenced by visual exploration strategy, motor speed, and individual task approach. Consistent with this distinction, SDMT was not significantly correlated with CTT-1 within the T2DM group after adjustment for age, sex, and years of education. Thus, SDMT may have been more sensitive to subtle white matter-related processing-speed vulnerability in this cohort. The absence of significant group differences in MMSE, RAVLT, CDT, and CTT-2 also warrants consideration. Although T2DM has been associated with episodic memory and executive dysfunction in previous studies, cognitive impairment in diabetes is heterogeneous and may vary according to disease stage, comorbidity burden, and test sensitivity. Although our T2DM group had suboptimal glycemic control and a moderate disease duration, the cohort excluded participants with dementia, stroke, major neurological disorders, and major psychiatric disorders, and showed relatively preserved global cognition, as reflected by the high mean MMSE scores. Therefore, the lack of significant RAVLT, CTT-2, CDT, and MMSE differences may reflect relatively mild diabetes-related cognitive involvement, ceiling effects on screening or less demanding tasks, insufficient sensitivity of the selected tests to subtle domain-specific deficits, or a cognitive phenotype dominated by processing-speed vulnerability rather than broad multidomain impairment. Consistent with this interpretation, exploratory regression analyses using non-SDMT cognitive measures as outcomes did not reveal consistent associations with the imaging markers.

Our findings contribute to the broader evidence linking brain clearance pathways with neurodegenerative and cerebrovascular disorders (47, 48). Experimental research indicates that factors such as vascular pulsatility, aquaporin-4 polarization, sleep, and neuroinflammation may modulate glymphatic function (49). In the cases of diabetes and aging, chronic low-grade inflammation, endothelial dysfunction, and BBB permeability have been associated with altered perivascular clearance pathways, accumulation of metabolic byproducts and white matter degeneration (19, 50, 51). In this regard, our data contribute to the existing literature by showing that, within a clinical T2DM population, MRI-derived markers associated with brain fluid regulation differ at the group level and correlate with CP morphology, WMH burden, and cognitive performance (41, 46).

Clinically, CP morphometry and FW mapping may provide complementary, noninvasive markers of diabetes-related brain alterations. CP volume can be derived from standard high-resolution T1WI, and FW mapping may capture fluid-related microstructural changes associated with WMH burden and cognitive performance. The combination of WMH and PVS quantification may help characterize risk for CSVD-related brain burden and cognitive vulnerability in T2DM. From a broader perspective, our results may encourage further longitudinal studies designed to test whether CP and FW alterations precede WMH progression or predict subsequent cognitive trajectories. Strategies like managing blood pressure, optimizing metabolism, improving sleep quality, and controlling inflammation may be biologically plausible targets for future studies of fluid-related brain alterations. MRI-based measurements such as FW fraction, WMH volume, and PVS volume may serve as candidate imaging outcomes in future interventional studies, although their therapeutic relevance remains to be established.

There are several limitations in this study that should be considered. First, since all measurements were obtained at a single time point, the temporal ordering of CP enlargement, FW changes, WMH progression, and cognitive decline cannot be established. The mediation models should be interpreted strictly as cross-sectional statistical indirect-effect analyses rather than evidence of mechanistic mediation, temporal ordering, or causal pathways. Longitudinal studies are needed to determine how these processes unfold over time. Second, the moderate-sized cohort from a single center needs to be replicated in larger, preferably multicenter samples. Although the *post-hoc* sensitivity power analysis suggested reasonable sensitivity to detect moderate overall effects in the multivariable regression models, smaller effects may not have been reliably detected. This is particularly relevant for the mediation analyses, in which several indirect effects were small in magnitude and proportional mediation estimates may be unstable in moderate-sized cross-sectional samples. Third, the neuropsychological battery might not fully capture all cognitive domains impacted by T2DM. SDMT was selected for downstream regression and mediation analyses because it was the only cognitive measure that survived FDR correction, rather than being prespecified as an *a priori* primary cognitive endpoint. This data-driven selection may introduce selective outcome reporting bias, and the SDMT-related findings should therefore be interpreted as exploratory. Future studies should prespecify primary cognitive outcomes and include more comprehensive domain-specific neuropsychological assessments. Fourth, the MRI measurements utilized in this study are indirect markers of glymphatic-related function and neuroinflammation rather than direct biological measurements. The FW fraction, for instance, may represent a mixture of interstitial fluid, vasogenic edema, and inflammatory modifications, whereas CP volume fails to capture cellular structure or functional state. Advanced MRI, molecular imaging, and CSF or blood biomarkers are necessary for validation. In addition, because PWMH volume was right-skewed, PWMH-related findings should be interpreted with caution. Sensitivity analyses using log-transformed PWMH volume suggested that PVS/FW–PWMH associations were relatively robust, whereas the CP–PWMH association was attenuated. Fifth, although all MRI scans were scheduled between 08:00 and 12:00 and participants with documented sleep disorders or sleep-modifying medication use were excluded, sleep duration, sleep quality, obstructive sleep apnea risk, and insomnia symptoms were not systematically assessed. Undiagnosed or subclinical sleep disturbance may still have differed between groups and could have influenced glymphatic-related measures, including FW fraction, ALPS index, and PVS volume. Future studies should incorporate standardized sleep assessments or objective sleep monitoring to better control this potential confounder. Finally, even after accounting for important demographic and clinical variables, there may still be confounding factors related to metabolism, medication, or lifestyle that need to be investigated in future studies with comprehensive phenotyping and sensitivity analyses. Although HbA1c and fasting blood glucose were incorporated into the revised regression models, these single-time-point measures may not fully capture cumulative glycemic exposure, glycemic variability, insulin resistance, hypoglycemic events, or long-term metabolic control. In addition, although insulin use was included as a diabetes-related covariate, we did not systematically adjust for other antidiabetic medication classes, such as metformin, GLP-1 receptor agonists, or SGLT2 inhibitors. These agents may have vascular, anti-inflammatory, or neuroprotective effects and could potentially influence FW fraction, WMH burden, or cognitive performance. Future studies with detailed medication histories and sufficient sample sizes should evaluate medication-specific effects using stratified or sensitivity analyses.

## Conclusion

In summary, our study showed concurrent differences and associations in CP volume, fluid-related white matter microstructure, periventricular WMH burden, and processing speed performance in T2DM. The observed associations among CP volume, FW fraction and PWMH burden suggests a fluid-related vulnerability of periventricular white matter in T2DM. These findings point to the relevance of fluid-related white matter alterations as potential imaging correlates of cognitive performance in diabetes. CP morphometry and FW imaging could be complementary non-invasive markers to characterize brain injury in T2DM. Longitudinal studies are required to determine whether these imaging markers precede WMH progression or cognitive decline.

## Data Availability

The raw data supporting the conclusions of this article will be made available by the authors, without undue reservation.
