# Supplementary material for: Free water–mediated associations among choroid plexus enlargement, white matter lesions, and cognitive performance in type 2 diabetes mellitus
Source: Front Endocrinol (Lausanne). 2026 Jun 18;17:1838132. doi: 10.3389/fendo.2026.1838132 (PMC13322795; doi:10.3389/fendo.2026.1838132)
Supplement: Supplementary file 1 [file Table1.docx]

Supplementary Material

Title: Free water–mediated associations among choroid plexus enlargement, white matter lesions, and cognitive performance in type 2 diabetes mellitus

**1. Supplementary sensitivity analysis using log-transformed normalized PWMH volume**

Because normalized PWMH volume showed a right-skewed distribution, sensitivity analyses were performed using log-transformed normalized PWMH volume. Log-transformed normalized PWMH volume was calculated as ln(normalized PWMH volume × 10⁴ + 1). After log transformation, the between-group difference in PWMH remained significant after adjustment for age, sex, and years of education (F = 7.13, P < 0.01, partial η² = 0.04). The corresponding T2DM-only regression analyses using log-transformed normalized PWMH volume as the outcome are shown in Supplementary Table 1.

Supplementary Table 1. Sensitivity regression analyses using log-transformed normalized PWMH volume as the outcome

| **Predictor** | **Model 1** | **Model 2** | **Model 3** |
| --- | --- | --- | --- |
| Model R² | 0.45 | 0.47 | 0.48 |
| Model P | < 0.001 | < 0.001 | < 0.001 |
| Normalized CP volume |  |  |  |
| β | 0.12 | 0.10 | 0.11 |
| P | 0.14 | 0.23 | 0.22 |
| PVS volume |  |  |  |
| β | 0.17 | 0.19 | 0.19 |
| P | 0.04 | 0.02 | 0.02 |
| FW fraction |  |  |  |
| β | 0.29 | 0.25 | 0.27 |
| P | < 0.01 | 0.01 | 0.01 |

Note. Regression analyses were performed within the T2DM group. The outcome was log-transformed normalized PWMH volume, calculated as ln(normalized PWMH volume × 10⁴ + 1). Values are standardized β coefficients and corresponding P values. Model 1 adjusted for age, sex, and years of education. Model 2 additionally adjusted for body mass index and vascular risk factors, including hypertension, hyperlipidemia, and current smoking. Model 3 further adjusted for insulin use, disease duration, HbA1c, and fasting blood glucose. Model R² and Model P refer to the overall regression model. CP, choroid plexus; FW, free water; PVS, perivascular space; PWMH, periventricular white matter hyperintensity; T2DM, type 2 diabetes mellitus.

**2. Supplementary exploratory regression analyses using non-SDMT cognitive measures as outcomes**

To examine whether the imaging–cognition associations extended beyond SDMT, exploratory regression analyses were additionally performed using non-SDMT cognitive measures as outcomes, including CTT-1, CTT-2, CDT, RAVLT immediate recall, and RAVLT delayed recall. In each model, normalized CP volume, FW fraction, PVS volume, PWMH volume, and DWMH volume were entered simultaneously as imaging predictors, with adjustment for age, sex, and years of education. These analyses did not reveal a consistent association pattern between the imaging markers and non-SDMT cognitive outcomes. The detailed results are shown in Supplementary Table 2.

Supplementary Table 2. Exploratory regression analyses within the T2DM group using non-SDMT cognitive measures as outcomes

| **Predictor** | **CTT-1** | **CTT-2** | **CDT** | **RAVLT**  **immediate** | **RAVLT**  **delay** |
| --- | --- | --- | --- | --- | --- |
| Model R² | 0.08 | 0.25 | 0.08 | 0.22 | 0.18 |
| Model P | 0.39 | < 0.01 | 0.40 | < 0.01 | 0.02 |
| Normalized CP volume |  |  |  |  |  |
| β | 0.07 | -0.01 | 0.06 | -0.04 | -0.12 |
| P | 0.55 | 0.91 | 0.59 | 0.69 | 0.29 |
| FW fraction |  |  |  |  |  |
| β | -0.05 | 0.02 | -0.01 | -0.08 | -0.03 |
| P | 0.69 | 0.89 | 0.94 | 0.49 | 0.85 |
| PVS volume |  |  |  |  |  |
| β | -0.17 | -0.18 | -0.09 | 0.05 | 0.18 |
| P | 0.12 | 0.08 | 0.42 | 0.61 | 0.09 |
| PWMH volume |  |  |  |  |  |
| β | -0.16 | 0.19 | -0.30 | -0.02 | -0.03 |
| P | 0.33 | 0.23 | 0.08 | 0.89 | 0.86 |
| DWMH volume |  |  |  |  |  |
| β | 0.24 | 0.06 | 0.43 | -0.07 | -0.16 |
| P | 0.11 | 0.69 | < 0.01 | 0.64 | 0.27 |

Note. Values are standardized β coefficients and corresponding P values. All models were adjusted for age, sex, and years of education. Normalized CP volume, FW fraction, PVS volume, PWMH volume, and DWMH volume were entered simultaneously as imaging predictors. These analyses were exploratory and were not used for primary inference. RAVLT, Rey Auditory Verbal Learning Test; CDT, Clock Drawing Test; CP, choroid plexus; CTT, Color Trails Test; DWMH, deep white matter hyperintensity; FW, free water; PVS, perivascular space; PWMH, periventricular white matter hyperintensity; SDMT, Symbol Digit Modalities Test. Model R² and Model P refer to the overall regression model including imaging predictors and covariates; only imaging predictors are shown in the table.
